# Investigation of *Escherichia coli* isolates from pigs and humans for colistin resistance in Lao PDR- a cross-sectional study

**DOI:** 10.1016/j.onehlt.2024.100745

**Published:** 2024-04-30

**Authors:** Vilaiphone Phomsisavath, Tamalee Roberts, Amphayvanh Seupsanith, Matthew T. Robinson, Phounsavanh Nammanininh, Somphaivanh Chanthavong, Vilada Chansamouth, Manivanh Vongsouvath, Watthana Theppangna, Peter Christensen, Stuart D. Blacksell, Mayfong Mayxay, Elizabeth A. Ashley

**Affiliations:** a Lao-Oxford-Mahosot Hospital-Wellcome Trust Research Unit (LOMWRU), Mahosot Hospital, Vientiane, Lao People’s Democratic Republic; b Centre for Tropical Medicine and Global Health, University of Oxford, United Kingdom; c Microbiology Laboratory, Mahosot Hospital, Vientiane, Lao People’s Democratic Republic; d National Animal Health Laboratory, Vientiane, Lao People’s Democratic Republic; e Mahidol Oxford Tropical Medicine Research Unit, Faculty of Tropical Medicine, Mahidol University, Bangkok, Thailand; f Institute of Research and Education Development (IRED), University of Health Sciences, Vientiane, Lao People’s Democratic Republic; g Lao One Health University Network (LAOHUN), Vientiane, Lao People’s Democratic Republic

**Keywords:** Antimicrobial resistance, One health, Colistin, *Escherichia coli*, Laos, Pigs

## Abstract

**Background:**

In Laos, colistin is not currently registered for use in humans. This One Health study aimed to estimate the prevalence of meat-producing pigs carrying colistin-resistant *Escherichia coli,* and investigate if *E. coli* causing invasive human infections were colistin-resistant.

**Methods:**

Between September 2022 and March 2023, rectal swabs were collected from 895 pigs from abattoirs in 9/17 Lao provinces. Pig rectal swabs and stored *E. coli* isolates from human blood cultures, submitted to Mahosot Hospital Microbiology laboratory between 2005 and 2022, were screened for colistin resistance on selective chromogenic agar with organism identification confirmed using MALDI-TOF MS. Suspected colistin-resistant isolates underwent colistin susceptibility testing by broth microdilution following European Committee on Antimicrobial Susceptibility Testing (EUCAST) guidelines. Isolates with MIC values of ≥2 μg/ml were tested for plasmid-mediated colistin resistance genes (*mcr-1*, *mcr-2,* and *mcr-3*) by multiplex SYBR Green PCR.

**Results:**

A total of 15/620 (2.41%) invasive human *E. coli* isolates were phenotypically colistin-resistant by broth microdilution (MIC values 4 to 8 μg/ml). The earliest isolate was from 2015 in a patient from Phongsaly province in Northern Laos. A total of 582/895 (65.02%) pig rectal swab samples contained colistin-resistant *E. coli*. The detected colistin resistance genes were predominantly *mcr-1* (57.8%, 346/598), followed by *mcr-3* (20.23%,121/598), and 22.24% (133/598) were found to co-harbour *mcr-1* and *mcr-3*. Among the 15 human isolates with colistin MIC values of ≥4 μg/ml, 12/15 were *mcr-1*.

**Conclusions:**

We found that colistin resistant *E. coli* is causing invasive infection in humans in Laos despite the fact it is not available for human use. Use in animals seems to be widespread, confirmed by high carriage rates of colistin-resistant *E. coli* in pigs. It is probable that food-producing animals are the source of colistin-resistant *E. coli* bloodstream infection in Laos, although these have been infrequent to date. This is a serious public health concern in the region that needs to be addressed by appropriate enforceable legislation.

## 1 Introduction

The World Health Organization (WHO) regards antimicrobial resistance (AMR) as one of the top ten global public health threats facing humanity [1]. Colistin (Polymyxin E) is one of the WHO Reserve (AWaRe) group of antibiotics used as a last resort option for the treatment of carbapenem-resistant Gram negative infections, especially in low-income settings where newer antibiotics for multidrug-resistant infections are either not registered or not affordable [[2], [3], [4]].

Colistin is used in food-producing animals, including pigs, as a treatment and as a growth promoter [5]. In 2015, the first plasmid-borne mobile colistin resistance gene (*mcr-1*) was detected in *Escherichia coli (E. coli)* from 78/523 (14.91%) samples of raw pork, 166/804 (20.64%) pigs, and in 16/1322 (1.21%) patients with infection in China [6]. Since then, an additional nine *mcr* genes have been described (*mcr-2* to *mcr-10*) [[7], [8], [9]]. Currently, colistin resistant *E. coli* has been reported from 54 countries on five continents worldwide. Asia has the highest reported burden, with most reports from China [10]. Asia and Europe have confirmed a wide diversity of *mcr* variants from various sample types. [10]. The most common plasmid-mediated colistin resistance genes in *Enterobacteriaceae* such as *E. coli, Salmonella enterica* serovar Typhi, and *Klebsiella pneumoniae* are *mcr-1*, *mcr-2,* and *mcr-3*, with high *mcr-*1 prevalence in *E. coli* in pigs. There have been no reports of *mcr-2* in *E. coli* in pigs in the Asia region [11].

The World Organization for Animal Health (WOAH) and WHO have stated that colistin cannot be used to prevent infections in animals, or as a first- or second-line treatment, unless no alternatives are available [12]. WOAH called for an urgent ban on the use of colistin, fluoroquinolones, and third and fourth-generation cephalosporins as growth-promoters in animals since these are essential drugs for the treatment of serious infections in humans [13,14].

Data on colistin resistance in bacterial isolates from human infections is sparse in many countries since standard disk-diffusion, or gradient minimum inhibitory concentration (MIC) methods are unreliable for antimicrobial susceptibility testing, related to poor solubility of colistin in solid agar [15]. Broth microdilution is preferred.

In Laos, colistin is not currently registered for human use [16]. However, there is evidence that colistin resistance has already emerged in the country [17]. Colistin used in animals is available in the form of water-soluble powder (e.g., Spira-Tylocol) for infectious disease treatment, and prevention in livestock [18]. There is a lack of information on the extent of usage of colistin in the animal sector in Laos. The first report of colistin resistance in *E. coli* in Laos was from faecal samples from 6/190 (3.15%) healthy humans, and 4/18 (22.22%) pigs collected in 2012 onwards, with suspected clonal transmission described from a 15 year old boy, and his pig from the same location although *mcr* genes were not tested for [19]. A study of 60 of each of human rectal swab samples, chicken faecal swabs, and chicken meat found that 9/60 (15%) of both people, and poultry faeces sampled were carrying colistin-resistant *E. coli*, 8/60 (13.33%) samples of chicken meat also contained colistin-resistant *E. coli*. PCR screening of *mcr-1* to *mcr-8* genes found 22/28 (78.57%) were *mcr-1* and 2/28 (7.14%) were *mcr-3.* Sequencing of selected isolates found the most common variant of genes was *mcr-1* (14.3%) with IncX4, IncI2, IncP1, IncFIA, and IncHI1 plasmids. Strains with chromosomal *mcr-1,* or plasmid-mediated *mcr-3* were also detected [20]. Furthermore, a study in 2018 described colistin-resistant *E. coli* contamination of human, animal, and environmental samples in Vientiane Capital, with 98/673 (14.56%) samples positive. These samples included human rectal swabs, chicken meat, chicken caeca, wastewater, flies, and dog faeces. Sequencing showed 82/673 (12.18%) isolates had *mcr-1,* 2/673 (0.29%) *mcr-3* and 14/673 (2.08%) had both *mcr-1,* and *mcr-3*. Sequencing of a subset of isolates found that the putative plasmid Inc. types associated with *mcr*-1 were IncX4, IncHI2, IncP1, IncI2, and IncFIA, and with *mcr*-3 were IncFII, IncFIA, IncFIB, IncP1, and IncR [21].

The Emergency Center for Transboundary Animal Diseases (ECTAD) of the Food and Agriculture Organization of the United Nations (FAO) has been working with the Department of Livestock and Fisheries (DLF) of the Ministry of Agriculture and Forestry (MAF) in Laos to develop a multisectoral strategy to tackle AMR which includes monitoring AMU in animals. To date, there have only been a few studies on colistin resistance in Laos in humans and animals and the full extent of the geographical distribution in various animal species is not known.

The aim of this study was to estimate the prevalence of colistin-resistant *E. coli* colonization in pigs being slaughtered for meat in multiple provinces in Laos, as well as the proportion of invasive human *E. coli* isolates in Laos that are colistin-resistant, and the earliest year of detection. Secondary objectives were to characterize the antimicrobial susceptibility of isolates from human invasive infections, and describe the associated colistin resistance genes (*mcr-1, mcr-2,* and *mcr-3*).

## 2 Material and methods

### 2.1 Sample collection

The study was conducted between September 2022, and March 2023. All available stored human *E. coli* isolates from all blood cultures from patients presenting with febrile illness submitted to Mahosot Hospital Microbiology laboratory in Vientiane (2005–2022) were included in the study, and their antimicrobial susceptibility data were extracted from the laboratory information management system. We performed a cross-sectional survey to collect rectal swabs from pigs before slaughter in 15 urban and rural abattoirs in nine provinces in Laos. These provinces were selected to represent the geographical range of Laos, and the abattoirs were selected after a pre-survey to determine which abattoirs slaughtered the highest number of pigs. One rectal swab was collected per pig, using a sterile Amies transport swab with charcoal (TRANSWAB®), by trained provincial veterinarians under the supervision of the National Animal Health Laboratory. Samples were stored at 4 °C and transferred to the Microbiology Laboratory, Mahosot Hospital for further processing, with a maximum transport time of 72 h. Data on pig demography (i.e., sex, age, breed), sampling site, sampling date, type of farm, and origin of pig were collected (Supplementary Material). Our target sample size for the pig survey was 100 per site which would enable us to detect a prevalence of 10% colistin resistance with 6% precision and 95% confidence.

### 2.2 Isolation and identification of colistin-resistant *E. coli*

Frozen stored human *E. coli* isolates from blood cultures were thawed and sub-cultured onto nutrient agar to ensure isolates were still viable before inoculating on Chromatic Colistin agar (Liofilchem®) to screen for resistance. Pig rectal swabs were placed in a 9 ml tube of Brain Heart Infusion broth with a colistin sulfate 10 μg disc (colistin sulfate CS 10 μg, Liofilchem®), and incubated at 35 ± 2 °C for five hours. After incubation, 25 μl were placed on the Chromatic Colistin agar, and streaked out, then incubated aerobically at 35 ± 2 °C for approximately 18–24 h. *E. coli* ATCC 25922 strain was used for quality control.

Colistin-resistant *E. coli* was identified by morphology and appearance with pink-reddish-mauve colonies on the selective Chromatic Colistin agar. Isolates were confirmed as *E. coli* by MALDI-TOF MS (VITEK MS, bioMérieux).

### 2.3 Colistin susceptibility testing

Confirmed *E. coli* isolates that grew on the Chromatic Colistin agar were tested for colistin susceptibility using the broth microdilution (BMD) method (ComASP™ Colistin, Liofilchem®) according to the manufacturer's instructions. A suspension of 0.5 MacFarland was added to Muller Hinton II broth and 100 μl was dispensed into the control well, and following wells (0.25–16 μg/ml). After incubating at 35 ± 2 °C for 16–20 h in ambient air, the lowest concentration of colistin that inhibited visible growth was read by the naked eye. The MICs were interpreted according to EUCAST guidelines version 12.0 (2022). The clinical breakpoint for defining colistin resistance is >2 μg/ml, but all isolates with an MIC of 2 μg/ml or greater were submitted for further testing.

### 2.4 Detection of *mcr* genes by the multiplex PCR assay

DNA was extracted from all *E. coli* isolates with a colistin MIC ≥2 μg/ml using the GeneJET Genomic DNA Purification Kit (Thermo Fisher Scientific Inc.), following the manufacturer's recommendations. PCR analysis for the presence of *mcr-1, −2* and *− 3* genes was carried out using a multiplex real-time PCR, based on a previously published assay [22]. Briefly, a 25 μl reaction mixture consisted of 1.25 U High Sensitivity Taq DNA Polymerase (PCRBIO), 0.3 μM each primer, and 1× SYBR Green. Reactions were run on Gentier 96E with the following thermal conditions: 95 °C for 2 min, followed by 40 cycles of 98 °C for 15 s, 60 °C for 15 s, 72 °C for 15 s.

### 2.5 Data analysis

Patient, and pig demographic details including age, sex, and province were included in the analysis. Farms were separated into large commercial, and small-scale traditional farms. Other antimicrobial susceptibility testing (AST) results were extracted from the laboratory database (disc diffusion method following contemporaneous Clinical and Laboratory Standard Institute (CLSI) until 2018, or EUCAST from 2018 to 2022 guidelines). All AST results were re-interpreted using EUCAST breakpoint tables version 12 (2022). Extended-spectrum β-lactamase (ESBL) confirmation test was done on isolates resistant to third-generation cephalosporins or cefpodoxime using the double-disc diffusion test (cefotaxime and ceftazidime with and without clavulanic acid). Statistical analyses were conducted using R Version 4.2.3 (2023/03/15). Data are reported in accordance with the STROBE checklist for cross-sectional studies (Supplementary data).

## 3 Results

From the Mahosot Microbiology laboratory records of 1000 stored *E. coli* isolates from blood cultures, we were able to find 620 isolates collected from 2005 to 2022. Patients came from 16 provinces in Laos with the majority from Vientiane Capital (457/620, 73.70%) where Mahosot Hospital is located. There were 403/620 (65%) female patients, and ages ranged from 1 day to 98 years old. A total of 895 rectal swabs from pigs were collected from 15 abattoirs in nine provinces. Data indicated that the province of origin of some pigs was different from the abattoir sites in provinces due to pigs being transported to abattoirs in provinces where the meat was to be sold so it remained fresh. There were seven provinces that transported pigs to slaughter in different provinces.

### 3.1 Culture results from colistin chromogenic agar

**Fig. 1:**
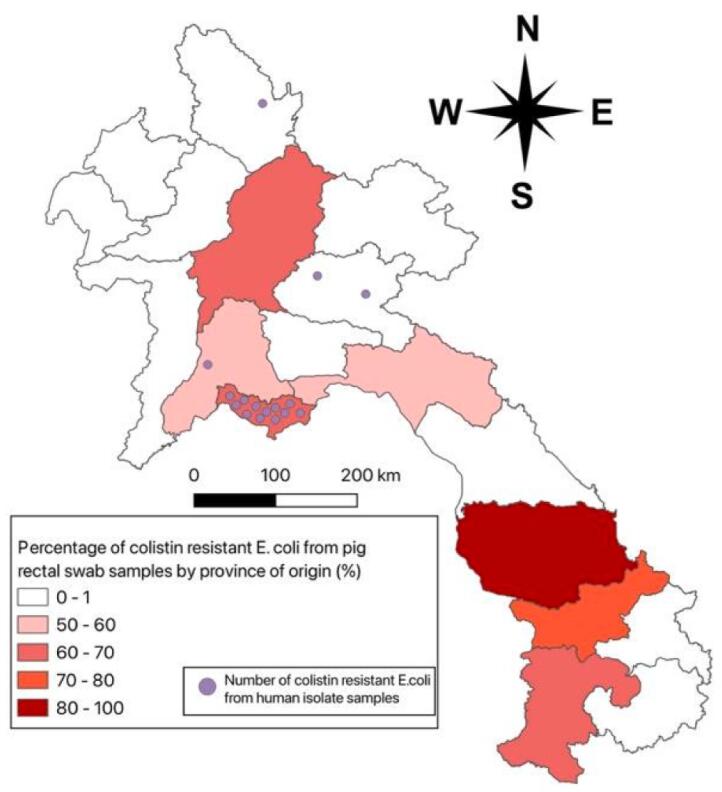
Map showing percentage of colistin-resistant (MIC ≥ 2 μg/ml) *E. coli* isolates from pig rectal swab samples by province of origin, and location of human invasive colistin-resistant *E. coli* bloodstream infections with detected *mcr* gene in Laos.

For the human blood culture isolates, 57/620 (9.19%) *E. coli* grew on the colistin chromogenic agar from patients aged between 2 days to 94 years old, of whom 39/57 (68.42%) were female. From the pig rectal swab samples, 582/895 (65.02%) grew on the colistin chromogenic agar. The percentage of samples with growth ranged from 18% (18/100 pigs originating from Luang Namtha) to 100% (100/100 pigs originating from Savannakhet) (Table 1, Fig. 1). The age of pigs ranged from 3 months to 2 years.

**Table 1:** Number of pig rectal swab samples collected, and percentage with colistin-resistant *E. coli* as shown by growth on colistin chromogenic agar and MIC >2 μg/mL, by site and farm type.

Province abattoir is located	Province pig originated from	Farm type	Number of samples from site	n (%) samples with colistin-resistant E.coli (defined as detection on chromogenic agar, and MIC > 2 μg/ml)
Bolikhamsay	Bolikhamsay	Large-commercial	40	24 (60%)
Bolikhamsay	Bolikhamsay	Small-scale	10	4 (40%)
Champasak	Champasak	Large-commercial	148	94 (63.51%)
Luang Namtha	Vientiane Province	Large-commercial	100	18 (18%)
Luang Prabang	Luang Prabang	Large-commercial	100	65 (65%)
Salavan	Champasak	Large-commercial	22	16 (72.72%)
Salavan	Salavan	Large-commercial	4	4 (100%)
Salavan	Salavan	Small-scale	23	16 (69.56%)
Savannakhet	Savannakhet	Large-commercial	100	100 (100%)
Vientiane Capital	Vientiane Province	Large-commercial	248	174 (70.16%)
Vientiane Province	Vientiane Province	Large-commercial	21	12 (57.14%)
Vientiane Province	Vientiane Province	Small-scale	29	24 (82.75%)
Xieng Khuang	Vientiane Capital	Large-commercial	50	31 (62%)

### 3.2 Phenotypic antimicrobial susceptibility testing

Microbroth dilution colistin susceptibility testing of *E. coli* that grew on the colistin chromogenic agar found 40/57 (70.17%) human *E. coli* isolates with an MIC <2 μg/ml and 17/57(29.82%) with an MIC ≥2 μg/ml. Two isolates had a MIC of 2 μg/ml (2/57, 3.50%), 7/57 (12.28%) had a MIC of 4 μg/ml, and 8/57 (14.03%) had a MIC of 8 μg/ml (Table 2). Among 11/17 isolates which had ESBL confirmatory testing, 10 were ESBL positive. All 12/17 isolates that were tested against meropenem were sensitive. AST results were re-interpreted using EUCAST v12.0 (2022) guidelines (Supplementary table 1). MIC values for the *E. coli* from pig samples that grew on the colistin chromogenic agar were 4 μg/ml (406/582, 69.75%), 8 μg/ml (157/582, 26.97%), 16 μg/ml (11/582, 1.89%) and > 16 μg/ml (8/582, 1.37%).

**Table 2:** Demographic and antimicrobial susceptibility testing information for the 17 human *E. coli* isolates with a colistin MIC of ≥2 μg/ml.

Patient No.	Age (years)	Year of sample	Gender	Province	Antimicrobial susceptibility test result				Colistin MIC (μg/ml)	Colistin resistance gene
					ESBL	Resistant	Sensitive			
1	54	2015	Female	Phongsaly	Negative	AMP, AMC, CAZ, CRO, CIP, C, CPD, SXT, TET	CN		8	*mcr-1*
2	60	2016	Male	Vientiane Capital	Not tested	AMP, SXT	AMC, CRO, CIP, CN		8	*mcr-1*
3	4 days	2016	Male	Vientiane Capital	Not tested	AMP	AMC, CRO, CIP, SXT, CN		4	*mcr-1*
4	54	2017	Female	Xieng Khuang	Positive	AMP, CAZ, CRO, CIP, C, CPD, SXT, TET, CN	AMC, AK, MEM		4	*mcr-1*
5	52	2018	Female	Vientiane Capital	Not tested	AMP, AMC	CRO, CIP, SXT, CN		8	*mcr-1*
6	32	2018	Female	Xieng Khuang	Positive	AMP, CAZ, CRO, C, CPD, SXT	AMC, CIP, AK, MEM, CN		8	*mcr-3*
7	2 days	2018	Female	Vientiane Capital	Not tested	AMP, SXT	AMC, CRO, CIP, CN		4	*mcr-1*
8	64	2019	Male	Vientiane Capital	Positive	AMP, AMC, CRO, CPD, SXT,	I* CIP	CAZ, C, AK, MEM, CN	4	*mcr-1*
9	50	2020	Male	Vientiane Capital	Positive	AMP, AMC, CAZ, CRO, CIP, CPD, SXT, AK, CN	C, MEM		4	*mcr-1*
10	94	2020	Female	Vientiane Capital	Not tested	AMP, SXT	AMC, CRO, CIP, CN		2	ND
11	74	2021	Female	Vientiane Capital	Not tested	AMP, AMC, SXT	CRO, CIP, CPD, FOX, AK, MEM, CN		2	*mcr-1*
12	77	2021	Male	Vientiane Capital	Positive	AMP, AMC, CRO, CIP, CPD, SXT, AK, CN	FOX, MEM		4	*mcr-1*
13	78	2021	Male	Vientiane Capital	Positive	AMP, CRO, CPD, SXT,	AMC, CIP, FOX, AK, MEM, CN		8	*mcr-1, mcr-3*
14	39	2021	Female	Vientiane	Positive	AMP, AMC, CRO, CIP, CPD, SXT, CN	FOX, AK, MEM		8	*mcr-1*
15	49	2022	Female	Vientiane Capital	Positive	AMP, AMC, CRO, CPD, SXT, CN	CIP, FOX, AK, MEM		8	*mcr-1, mcr-3*
16	90	2022	Male	Vientiane Capital	Positive	AMP, AMC, CRO, CIP, CPD, SXT, CN	FOX, AK, MEM		8	*mcr-1*
17	75	2022	Male	Vientiane Capital	Positive	AMP, AMC, CRO, CIP, CPD, SXT, CN	FOX, AK, MEM		4	*mcr-1*

AMP = Ampicillin 10 μg, AMC = Amoxicillin- clavulanic acid 30 μg, CAZ = Ceftazidime 10 μg, CRO = Ceftriaxone 30 μg, CIP = Ciprofloxacin 5 μg, C = Chloramphenicol 30 μg, CPD = Cefpodoxime 10 μg, SXT = Trimethoprim- Sulfamethoxazole 25 μg, TET = Tetracycline 30 μg, FOX = Cefoxitin 30 μg, AK = Amikacin 30 μg, MEM = Meropenem 10 μg, CN = Gentamicin10μg, I* = Susceptible, increased exposure, ND = Not detected.

### 3.3 Molecular detection of colistin-resistance genes

The *mcr* genes identified in the human *E. coli* isolates with a colistin MIC of >2 μg/ml were predominantly *mcr-1* 13/15 (86.67%), with 1/15 (6.67%) *mcr-3*, and 2/15 (13.33%) with both *mcr-1,* and *mcr-3* genes. For the two isolates (2/620, 0.32%) that had a MIC of 2 μg/ml, one (50%) was *mcr-1* positive while the other tested negative by PCR for *mcr-*1-3. From the pig rectal swabs 333/582 (57.21%) were *mcr-1*, 118/582 (20.27%) were *mcr-3*, and 131/582 (22.50%) were both *mcr-1,* and *mcr-3.* There were no *mcr-2* detected from either the human or pig isolates.

## 4 Discussion

This One Health study described the prevalence of colistin-resistant *E. coli* in human invasive isolates, and pig carriage isolates in Laos. Antimicrobial resistance is increasing in the country with >50% of *E. coli* isolates from bloodstream infections diagnosed in a central hospital confirmed as ESBL-producers in 2023 [18]. This is fuelling the use of meropenem and carbapenem resistance has emerged in recent years [23] which will necessitate using colistin for treatment in the near future. Although colistin is not currently available in Laos in the human health sector, 2.42% (15/620) of invasive human *E. coli* isolates were found to be colistin-resistant in this study. Two earlier studies in Laos found a prevalence of 15–45% of colistin-resistant *E. coli* from human rectal swabs but the prevalence in invasive infections has not been described previously [20,21]. The first colistin-resistant isolate from this study was from 2015 from a patient from Northern Laos, which borders China. Since 2015 the number of colistin-resistant *E. coli* isolates has increased but remains relatively low in our centre. Most patients (11/15, 73.33%) came from Vientiane Capital, which is the main catchment area for Mahosot Hospital, with no positive results from 12 patients from the southern provinces of Laos, despite very high carriage rates in pigs. Colistin-resistant *E. coli* was found in 65.02% (582/895) of pig rectal samples. The highest percentage (100%, 100/100) of colistin resistance in *E. coli* from pigs was seen in samples from Savannakhet in the south of Laos as shown in Fig. 1.

The *mcr-1* gene was the most common gene detected in this study (57.86%, 346/598), which has been reported from most studies around the world [10]. Isolates co-harboring *mcr-1* and *mcr-3* genes were seen in 22.24% (133/598) of isolates which has also been found in several studies [17]. There were no *mcr-2* genes detected which is similar to other studies from the region. The majority of reports of *mcr-2* come from Europe [24], Egypt [25], and China [26]. Interestingly, one of the two isolates with a MIC of 2 μg/ml from this study was positive for *mcr-1*. In a previous study in Vietnam, *E. coli* isolates from humans, animals and the environment were screened for *mcr*, irrespective of the colistin MIC results. They found 60.63% (97/160) of *mcr-1* carrying *E. coli* isolates were phenotypically susceptible to colistin with a MIC of ≤2 μg/ml [27]. How the presence of *mcr* with MIC <2 μg/ml correlates with clinical response to treatment is unclear but suggests clinical breakpoints may need to be reviewed.

We do not know whether the higher percentage of colistin-resistant *E. coli* from pig rectal swabs from small and large farms in this study (65.02%, 582/895) compared to neighboring countries with 41.64% (112/269) colistin-resistant *E. coli* reported from pigs in small-scale farms in Thailand [28], and 45.69% (53/116) small and large pig farms in Vietnam is as a result of different antimicrobial usage in the different countries or different sampling strategies [29].

Ten human colistin-resistant isolates were ESBL-positive, and six were also resistant to ciprofloxacin and gentamicin, although all retained susceptibility to meropenem. This multidrug class resistant phenotype is typical of most ESBL-producing *E. coli* bloodstream infections in Laos, necessitating that almost all affected patients are treated with meropenem. Carbapenem resistance in Enterobacterales is starting to emerge and colistin is the last line antibiotic needed to treat infections caused by these organisms. Newer antibiotics are largely unaffordable and inaccessible in most low resource settings currently.

There is increasing demand for pork and poultry meat in Lao PDR (Laos), with pork supply of around 15.23 kg per capita per year since 2015, according to FAOSTAT data. A high-level cross-sectoral collaboration involving the WHO, the Food and Agriculture Organization, the Ministry of Health, the Ministry of Agriculture and Forestry, and other key stakeholders in Laos has been developing the national strategic plan to tackle the issue of AMR with a key priority action to improve the awareness and understanding of AMR in the country [30]. More data on colistin consumption and usage in the animal sector would be useful. There also needs to be better engagement with farmers and the public to increase understanding of AMR, and the role of antimicrobial use as a major driver of AMR.

Limitations of this study include our inability to prove animal-to-human transmission of colistin-resistant *E. coli* to confirm the likely role of the food chain, and in particular pigs or pig meat, in causing infection. Whole-genome sequencing may have provided stronger evidence of pig to human transmission but we did not have the resources to perform this. The number of samples collected was either capped at the target number, or was all pigs that were slaughtered which did not always meet our target. Therefore, our sample may not be representative of pigs across the whole country. Although there were human isolates from 16 provinces, the majority came from Vientiane Capital. As *mcr* genes 1–3 are the most commonly detected in the region, we only tested for those, but it is possible that other genes may have been in the samples. Finally, we were not able to determine what food the pigs were given and if it included colistin.

## 5 Conclusions

In this study we described colistin resistance in *E. coli* in pigs and humans in Laos. We confirmed that colistin-resistant *E. coli* is a cause of invasive infections in humans in Lao PDR (2.42%, 15/620), and carriage is widespread in more than half (65.02%) of pigs from both small and large farms. The most likely driver is the use of colistin as a growth-promoter in the animal sector. Further research could help confirm the main reservoirs of resistance in humans, and routes of transmission. The findings of this study can be used to advocate for a robust antimicrobial stewardship program to minimize unnecessary use of colistin within animal sector, and to reserve colistin as a treatment for critically ill patients with carbapenem-resistant infections.

## Ethics committee approvals

Ethics committee approval for use of data associated with human isolates was granted by the Oxford Tropical Research Ethics Committee (OxTREC Ref: 0325/FMS/2002; 006–27; 41–20), and the Lao National Ethics Committee for Health Research (NECHR). Ethical approval for research involving animals was approved by the Department of Livestock and Fisheries Institutional Review Board (IRB) of Animal Health, Ref. No.: 2525/DLF.IRB. Permission to take rectal swabs was granted by the Department of Livestock and Fisheries through the National Animal Health Laboratory and the Lao PDR-Cambodia-Thailand Veterinary Laboratory Capacity Building Project (LACATH4).

## Funding

This research was funded by 10.13039/100010269Wellcome (grant number: 220211/Z/20/Z). EA, TR, MTR, and SDB are funded by the 10.13039/100010269Wellcome Trust of the United Kingdom. VP was funded by the SEAOHUN Fellowship Program with the generous support of the American people through the US Agency for International Development (USAID) One Health Workforce - Next Generation (OHW-NG) Award 7200AA19CA00018. The project or effort depicted was or is sponsored in part by the Department of Defense, Defense Threat Reduction Agency. The content of the information does not necessarily reflect the position or the policy of the federal government, and no official endorsement should be inferred. For the purpose of Open Access, the author has applied a CC BY public copyright license to any Author Accepted Manuscript version arising from this submission.

## CRediT authorship contribution statement

**Vilaiphone Phomsisavath:** Writing – original draft, Project administration, Methodology, Formal analysis, Data curation. **Tamalee Roberts:** Writing – review & editing, Validation, Supervision, Investigation. **Amphayvanh Seupsanith:** Methodology. **Matthew T. Robinson:** Writing – review & editing, Validation, Investigation, Conceptualization. **Phounsavanh Nammanininh:** Methodology. **Somphaivanh Chanthavong:** Methodology. **Vilada Chansamouth:** Writing – review & editing, Validation, Investigation. **Manivanh Vongsouvath:** Writing – review & editing, Validation, Investigation. **Watthana Theppangna:** Writing – review & editing, Validation, Supervision, Investigation. **Peter Christensen:** Writing – review & editing, Validation, Investigation, Conceptualization. **Stuart D. Blacksell:** Writing – review & editing, Validation, Supervision, Investigation. **Mayfong Mayxay:** Writing – review & editing, Validation, Supervision, Investigation. **Elizabeth A. Ashley:** Writing – review & editing, Validation, Supervision, Investigation, Conceptualization.

## Declaration of competing interest

The authors declare that there are no conflicts of interest.

## Data Availability

All data are included in the submitted text and tables. Data are available on request from the MORU Data Access Committee (datasharing@tropmedres.ac). For the purpose of Open Access, the author has applied a CC BY public copyright license to any Author Accepted Manuscript version arising from this submission. The contents and associated materials are the responsibility of the authors, and do not necessarily reflect the views of USAID, or the US Government.

## Appendix A Supplementary data

Supplementary material Image 1
