# Supplementary material for: Investigation of Escherichia coli isolates from pigs and humans for colistin resistance in Lao PDR- a cross-sectional study
Source: One Health. 2024 Apr 30;18:100745. doi: 10.1016/j.onehlt.2024.100745 (PMC11079391; doi:10.1016/j.onehlt.2024.100745)
Supplement: Supplementary file 1 — Supplementary material [file mmc1.docx]

**SUPPLEMENTARY MATERIAL**

**PCR methods**

PCR was performed using a previously described assay [1], with minor adjustments. PCR was performed in a 25 µl reaction mixture containing 1x PCRBIO reaction buffer (PCR Biosystems Ltd, London, UK), 0.3 µM of each primer for detection of *mcr-1*, *-2* and *-3* genes, 1x SYBR Green I, 1.25U of PCRBIO HS Taq DNA Polymerase and 2 µl of template DNA. All assays were run with positive and negative (water) controls. PCRs were performed with the following cycling conditions: initial denaturation at 95°C for 2 minutes, followed by 40 cycles of denaturation at 95°C for 15 seconds, annealing at 60°C for 15 seconds, and elongation at 72°C for 15 seconds. To determine the *mcr* gene present, PCR products were run on a melt curve from 72°C to 95°C to determine melting temperature (T_m_). Results were confirmed by viewing PCR product size by gel electrophoresis on a 2.0% agarose gel. Mean T_m_ for *mcr-1* isolates was 86.5°C, and 79.5°C for *mcr-3*.

**Supplementary table 1. Sequence of primers were used for PCR.**

| **Gene** | **Forward primers (5’-3’)** | **Reverse primers (3’-5’)** |
| --- | --- | --- |
| mcr-1 | AAAGACGCGGTACAAGCAAC | GCTGAACATACACGGCACAG |
| mcr-2 | CGACCAAGCCGAGTCTAAGG | CAACTGCGACCAACACACTT |
| mcr-3 | ACCTCCAGCGTGAGATTGTTCCA | GCGGTTTCACCAACGACCAGAA |

Reference:

[1] J. Li, X. Shi, W. Yin, Y. Wang, Z. Shen, S. Ding, S. Wang, A multiplex SYBR green real-time PCR assay for the detection of three colistin resistance genes from cultured bacteria, feces, and environment samples, Front Microbiol 8 (2017). https://doi.org/10.3389/fmicb.2017.02078.

**Supplementary table 2.** **Antimicrobial susceptibility testing zone sizes, and updated interpretation using EUCAST v12.0 2022 guidelines for the 17 human *E. coli* isolates with a colistin MIC of ≥ 2 μg/ml.**

|  | **AMP** | | **AMC** | | **CAZ** | | **CRO** | | **CIP** | | **C** | | **CPD** | | **SXT** | | **TET*** | | **FOX** | | **AK** | | **MEM** | | **CN** | |
| --- | --- | --- | --- | --- | --- | --- | --- | --- | --- | --- | --- | --- | --- | --- | --- | --- | --- | --- | --- | --- | --- | --- | --- | --- | --- | --- |
| Patient 1 | 6 | R | 10 | R | 17 | R | 19 | R | 6 | R | 6 | R | 6 | R | 6 | R | 6 |  |  |  | 20 | S | 28 | S | 20 | S |
| Patient 2 | 6 | R | 18 | R |  |  | 30 | S | 25 | S |  |  |  |  | 6 | R |  |  |  |  |  |  |  |  | 17 | S |
| Patient 3 | 6 | R | 18 | R |  |  | 27 | S | 21 | R |  |  |  |  | 21 | S |  |  |  |  |  |  |  |  | 18 | S |
| Patient 4 | 6 | R | 20 | S | 17 | R | 6 | R | 6 | R | 6 | R | 6 | R | 6 | R | 6 |  |  |  | 21 | S | 30 | S | 6 | R |
| Patient 5 | 6 | R | 13 | R |  |  | 29 | S | 29 | S |  |  |  |  | 25 | S |  |  |  |  |  |  |  |  | 21 | S |
| Patient 6 | 6 | R | 19 | S | 13 | R | 6 | R | 21 | R | 6 | R | 6 | R | 6 | R |  |  |  |  | 18 | S | 27 | S | 17 | S |
| Patient 7 | 6 | R | 21 | S |  |  | 31 | S | 31 | S |  |  |  |  | 6 | R |  |  |  |  |  |  |  |  | 20 | S |
| Patient 8 | 6 | R | 18 | R | 24 | S | 6 | R | 24 | I | 28 | S | 6 | R | 6 | R |  |  |  |  | 20 | S | 31 | S | 20 | S |
| Patient 9 | 6 | R | 15 | R | 13 | R | 8 | R | 6 | R | 25 | S | 6 | R | 6 | R |  |  |  |  | 19 | S | 31 | S | 9 | R |
| Patient 10 | 6 | R | 19 | S |  |  | 31 | S | 27 | S |  |  |  |  | 6 | R |  |  |  |  |  |  |  |  | 21 | S |
| Patient 11 | 6 | R | 12 | R |  |  | 26 | S | 27 | S |  |  | 21 | S | 6 | R |  |  | 21 | S | 18 | S | 27 | S | 17 | S |
| Patient 12 | 6 | R | 11 | R |  |  | 6 | R | 6 | R |  |  | 6 | R | 6 | R |  |  | 22 | S | 16 | R | 27 | S | 6 | R |
| Patient 13 | 6 | R | 20 | S |  |  | 6 | R | 25 | S |  |  | 6 | R | 6 | R |  |  | 26 | S | 21 | S | 29 | S | 20 | S |
| Patient 14 | 6 | R | 17 | R |  |  | 10 | R | 21 | R |  |  | 6 | R | 6 | R |  |  | 22 | S | 19 | S | 31 | S | 6 | R |
| Patient 15 | 6 | R | 17 | R |  |  | 6 | R | 27 | S |  |  | 6 | R | 6 | R |  |  | 24 | S | 21 | S | 28 | S | 6 | R |
| Patient 16 | 6 | R | 13 | R |  |  | 6 | R | 6 | R |  |  | 6 | R | 6 | R |  |  | 24 | S | 20 | S | 31 | S | 11 | R |
| Patient 17 | 6 | R | 17 | R |  |  | 9 | R | 21 | R |  |  | 6 | R | 6 | R |  |  | 22 | S | 18 | S | 28 | S | 6 | R |

R = Resistant, I = Intermediate, S = SAMP = Ampicillin 10μg, AMC = Amoxicillin- clavulanic acid 30μg, CAZ = Ceftazidime 10μg, CRO = Ceftriaxone 30μg, CIP = Ciprofloxacin 5μg, C = Chloramphenicol 30μg, CPD = Cefpodoxime 10μg, SXT = Trimethoprim- Sulfamethoxazole 25μg, TET = Tetracycline 30μg, FOX = Cefoxitin 30μg, AK = Amikacin 30μg, MEM = Meropenem 10μg

*There is no interpretation for Tetracycline in the EUCAST v12.0 guidelines.

**Supplementary table 3.** **Demographic data of pigs carrying *E. coli* with a colistin MIC of ≥ 2 μg/ml.**

|  | **n (%) samples with colistin-resistant *E. coli*** |
| --- | --- |
| **Sex** | |
| Male | 287/466 (61.58%) |
| Female | 295/429 (68.76%) |
| Total | 582/895 (65.02%) |
| **Age** | |
| 3-5 months | 296/470 (62.97%) |
| 6-8 months | 251/366 (68.57%) |
| 1-2 years | 35/59 (59.32%) |
| Total | 582/895 (65.02%) |
| **Breed** | |
| Large White pig | 158/248 (63.70%) |
| Three Blood Hybrid | 100/100 (100%) |
| Native Pig | 9/16 (56.25%) |
| Mix breed | 36/50 (72%) |
| Meat pig breed | 201/281 (71.53%) |
| Hybrid pig | 78/200 (39%) |
| Total | 582/895 (65.02%) |
